# The economic context of pursuing online medication abortion in the United States

**DOI:** 10.1016/j.ssmqr.2021.100003

**Published:** 2021-08-27

**Authors:** Dana M. Johnson, Melissa Madera, Rebecca Gomperts, Abigail R.A. Aiken

**Affiliations:** a The University of Texas at Austin, USA; b Aid Access & Women on Web, Amsterdam, the Netherlands

**Keywords:** Self-managed abortion, Self-induced abortion, Medication abortion, Telemedicine, Financial hardship

## Abstract

Access to in-clinic abortion has become increasingly restricted in the U.S. and for many, the high cost of care is a significant barrier. However, little is known about how financial circumstances shape the alternate pathways to abortion care people seek when the clinic is out of reach. In a unique sample of people who used medication abortion pills from Aid Access, a non-profit telemedicine service, we examine the impact of economic circumstances on abortion care pathway decision-making and experiences seeking care. Between June and August 2019, we conducted 80 anonymous, semi-structured in-depth interviews with U.S. residents who self-managed their own abortions using medication abortion pills from Aid Access. Participants were asked about their experiences seeking abortion, and their motivations for using the service. We coded interviews using an iteratively developed coding guide and performed thematic analyses to identify key themes. The unaffordable cost of in-clinic abortion was a key reason why participants sought care using online telemedicine. Experiences of personal financial hardship exacerbated by restrictive policies impacted participants’ ability to access the clinic. For participants with children, their financial decisions were further guided by the concerns of providing economic stability for their family. Although telemedicine was considered more affordable than in-clinic care, for some, the suggested donation of $90 still posed a financial burden and accessing pills at no cost or a reduced cost was necessary. The availability of affordable telemedicine and policy interventions addressing Medicaid and insurance coverage for abortion would democratize abortion access for populations with low incomes.

## 1. Introduction

For those seeking to end a pregnancy, the cost of in-clinic abortion care can be a significant barrier. In 2014, three quarters of abortion patients in the United States were poor or low-income, and 49% lived below the federal poverty level (Jerman, Jones, & Onda, 2016). In-clinic medication abortion care costs range from $250 to $2,000 (Witwer et al., 2020), with the majority of patients paying out of pocket for care (Jerman et al., 2016a). While abortion funds are able to help cover the costs of some, they are unable to help everyone in need of assistance (Ely et al., 2016). People who struggle to afford care delay or forgo paying necessary bills such as rent, food, or utilities to pay for their abortion (Jones, Upadhyay, & Weitz, 2013), and lack of affordable access is linked to ongoing personal financial distress, debt, and poor credit (Miller et al., 2020).

Restrictive abortion laws in the U.S. add further economic burdens to clinic access. Between 2011 and 2017, 401 abortion restrictions were enacted in U.S. states, and 29 states have policy climates deemed hostile or extremely hostile to abortion rights (Nash et al., 2016a, 2016b). The Hyde Amendment, which bans federal insurance plans from paying for abortion care, presents a major financial obstacle for patients (Henshaw et al., 2009). Mandatory waiting periods contribute to delay in patient care, increasing the price of the procedure (Guttmacher Institute, 2020), and state-directed counseling add additional costly appointments (Joyce et al., 2009). Targeted Regulation of Abortion Provider (TRAP) laws have closed clinics, necessitating travel that involves out-of-pocket costs such as childcare, lodging, transportation, and lost wages (Gerdts et al., 2016; Miller et al., 2020, p. 13; Jones et al., 2013; Jerman et al., 2017). In extreme circumstances, the requirement to travel forces people to delay or miss their abortion (Upadhyay et al., 2014; White et al., 2016). These restrictive laws result in significant increases in cost for care and overall worsened economic outcomes for people seeking an abortion (Foster et al., 2018).

As abortion becomes increasingly restricted, researchers have found evidence of some people in the U.S. forgoing the clinic altogether and self-managing their abortion on their own, outside of the formal healthcare setting (Jones, 2011). People have attempted self-management using a variety of methods, such as herbs, teas, homeopathic remedies, or self-harm (Moseson et al., 2020). However, with the proliferation of information-sharing on the internet came expanded access to abortion medications misoprostol and mifepristone (Kerestes et al., 2019), and self-managed abortion has recently manifested in the use of these medications ordered from the internet (Aiken et al., 2020a, 2020b). In the international context, studies have examined how the non-profit online telemedicine provider, Women on Web, has provided medical consultation, medication, and self-management guidance to people in the Republic of Ireland, Northern Ireland (Aiken et al., 2016a, 2017), Latin America (Aiken et al., 2016b), and Great Britain (Aiken et al., 2018b), and to U.S. service members stationed throughout the world (Fix et al., 2019; Grindlay et al., 2011).

In 2018, a new online telemedicine service called Aid Access became the first service to provide consultation and medication abortion in the United States, revolutionizing abortion access by assisting U.S. residents with self-management (Aid Access. Available at:, 2020). There is a growing literature on the knowledge of and interest among U.S. residents to use online medication for self-managing their own abortions (Aiken et al., 2020a, Aiken, Broussard, Johnson, & Padron, 2018a), but little is known about the factors motivating some people to use this service as an alternative to the clinic, including the role that their personal economic context might play.

### 1.1. Study purpose

The purpose of this study was to examine a unique sample of U.S.-based individuals who obtained self-managed medication abortion from Aid Access, the sole nationwide online telemedicine service in the United States. Specifically, we sought to explore: What role do socioeconomic factors play in an individual’s decision to self-manage using online telemedicine?; And, what impact does an individual’s economic situation have on their experience finding and engaging with such a pathway?

## 2. Materials and methods

### 2.1. Recruitment and data collection

Between June and August of 2019, we conducted anonymous in-depth interviews with 80 individuals living in the U.S. who accessed self-managed medication abortion using Aid Access. All interviewers were trained researchers with previous experience conducting in-depth interviews and qualitative analysis. Aid Access provides the abortion medications mifepristone and misoprostol according to the World Health Organization approved protocol up to 10 weeks of gestation (The World Health Organization, 2014). To acquire medication, individuals fill out an online consultation form with their gestational age, medical history, and demographic characteristics. After their eligibility is determined, a physician prescribes mifepristone and misoprostol, which is shipped directly to the individual. An email providing a step-by-step guide for self-managing at home, including what to expect, and signs of potential complications is sent along with the medications, and individuals have access to support from an online helpdesk throughout the process. Those accessing the service are requested to make a donation of $90, which may be reduced or waived depending on individual circumstances.

Participants were recruited by email invitation, which was sent by the organization to all who accessed the service between May and August of 2019. To be eligible to participate individuals had to be at least 18 years old. To preserve anonymity, all interviews were audio only, and interested individuals contacted the research team to arrange an interview using an encrypted texting App that allows both the participant and the interviewer to remain anonymous. To maintain privacy participants communicated exclusively through the App and did not share any potentially identifying information. Trained research team members also used the App to conduct the interviews, and participants were assigned pseudonyms. All participants gave their informed consent to participate and for the interview to be recorded. We stored audio recordings on a protected server and deleted them from any other location immediately after the interview. Interviews lasted between 27 and 118 min. At the conclusion of the interview, participants received a digital $90 gift card in appreciation for their time. Interviews were conducted using a semi-structured interview guide, based upon the chosen research questions and informed by previous studies of self-managed medication abortion (Aiken et al., 2018a). Non-identifying geographic and demographic characteristics were collected at the conclusion of each interview. Immediately after the conversation ended, researchers made field notes regarding key content, including the circumstances and demographics of participants. The study was reviewed and approved by the Institutional Review Board at the University of Texas.

### 2.2. Analysis

Interviews were transcribed verbatim by an in-house transcription service. Prior to transcription, all audio recordings were reviewed for any potentially identifying information and edited by the research team to maintain confidentiality. To pilot the interview guide we conducted several test interviews to refine the instrument. The final sample size of 80 interviews was decided when the research team determined a balance between thematic saturation and available resources had been achieved. Drawing from grounded theory techniques and principles, our team collectively developed an initial coding guide for analysis using the constant comparative method, and engaging in memo writing and regular discussions of potential themes (Charmaz, 2014a, 2014b; Creswell & Poth, 2018a, 2018b). Each interview was then coded by a member of the research team using thematic analysis, allowing for the development of new themes from the data (Charmaz, 2014a, 2014b). To ensure consistency among coders, interviews were coded independently by two members and then compared to check for possible discrepancies (Boyatzis, 1998). Interviews were coded and organized using Dedoose 8.3.20 software. Guided by our research questions, we then conducted a second round of coding utilizing axial coding methods to capture subcategories related to the core category of financial barriers and economic hardship (Creswell & Poth, 2018a, 2018b).

## 3. Findings

### 3.1. Sample demographics

Participants were diverse with respect to demographic characteristics (Table 1). The age range of participants was 18–44 years, and 54% were in their late 20’s or early 30’s. White participants made up 46.3% of the sample, 15% were Hispanic, 17.5% were Black or African American, 6.3% were Mixed Race, 3.8% were White and Hispanic, 2.5% were Mixed Race Hispanic, 2.5% were Native Hawaiian, 1.3% were Asian, 1.3% were Native American, 1.3% were Middle Eastern, 1.3% self-identified as “other,” and 1.3% declined to answer. Most participants (81.3%) were heterosexual, 11.3% were bisexual, 2.5% were pansexual, 1.3% were “bicurious,” 1.3% were “no preference,” and 2.5% declined to answer. Almost all participants (98.8%) were female, and 1.3% were non-binary. Mothers made up 61.3% of our sample, with the number of children ranging from one to three. The employment status of participants varied, and 52.5% were working full time at the time of their abortion, 21.3% were working and in school, 18.8% were unemployed, 6.3% were in school full-time, and 1.3% were in school and retired.

Participants were also diverse in terms of their geographic location, and thus their state-based reproductive health policy context (Table 2). We used the Guttmacher Institute’s classification of state-based abortion policy context to categorize states as “very hostile,” “hostile,” “middle ground,” and “supportive.” These groups reflect six categories of restrictive abortion policies and six categories of supportive abortion policies. Based on the number of policies in each group, a state is placed into a policy classification (Guttmacher Institute. (20, 2019). Hostile states were the most common state of residence (55%), and 30% resided in very hostile states, 12.5% in supportive states, 2.5% did not disclose their state, and no participants resided in a middle ground state. Geographically, 43.8% of participants lived in a city, 17.5% lived in a suburb, 18.8% lived in a town, 17.5% in a rural area, and 2.5% declined to answer.

### 3.2. Thematic analysis

Five key themes emerged from our analysis: 1) when participants first sought abortion care, many immediately looked for a clinic, but shifted perspective after learning of the high cost of in-clinic care; 2) after ruling out the clinic, participants described how circumstances of personal financial hardship were a key motivator for pursuing the pathway of online telemedicine; 3) participants described how restrictive abortion policies added further costs to in-clinic care; 4) navigating the economic pressures of motherhood and providing for their families added an additional economic calculus for mothers; and 5) although online telemedicine was significantly cheaper than the clinic there was still some cost associated with telemedicine, and for some, even a little was too much.

### 3.3. Consideration and cost of in-clinic care

When participants first decided to have an abortion, many considered the clinic as an option for care. Participants either called or visited the clinic to begin the process, and while gathering information, participants expressed shock at the price. As Treecie, a 32-year-old Oklahoma woman explained: “*I searched for clinics in my area and I found two, one of them was relatively close and the other one was about an hour away. Both of the clinics…it was expensive, close to a thousand dollars. And it was ridiculous to me because I knew that I was only (a) few weeks*.”

For the majority of the women we spoke with, the cost of the clinic was impossible to financially manage. Fiona, a 30-year-old mother in Pennsylvania, received a $600 quote from Planned Parenthood and immediately searched online “*to find a cheaper method*.” Beth, a 28-year-old Louisiana woman explained: “*You can’t even get an appointment to visit if you don’t have $400 or something like that. And then you have to come with an additional check on the day of, and depending on how many weeks you are, it’s an additional hundred per week or something like that. It’s like, no way can I afford that being unemployed and trying to make rent, you know?*” Joelle, a 25-year-old Kentucky woman, said once she was quoted the price for the clinic, she thought: “*there’s no way I can afford the $500, [and] it was either that or just have the baby. I ultimately thought if it doesn’t work, I guess I’ll just have the baby*.” Beth and Joelle’s experiences, in particular, reflect an awareness of the cost of in-clinic abortion care, and the acute necessity to find a more affordable option. This theme is reflected throughout our sample, prompting participants to seek an alternative pathway to care.

### 3.4. Financial hardship as a motivator for seeking online telemedicine

After ruling out the clinic, participants described experiences of personal financial hardship as central to their decision to pursue online telemedicine as the best alternative for affordable care. People described experiences of being low-income, uninsured, experiencing sudden economic instability, and living paycheck-to-paycheck, as impacting how they decided to have an abortion. Rosie, a 23-year-old mother in Oklahoma who was preparing to return to school in the fall, put it simply: “*I am going through financial hardship right now…I knew at that point…I’m never going to be able to afford it because they quoted me about $1000*.” After ruling out the clinic, she tried self-managing using ascorbic acid, black and blue cohosh, and contacted a reiki healer. When these methods did not work, she confided in a friend who showed her the online telemedicine website: “*I told her that I was desperate, afraid and she said, ‘I understand,’ and showed me what to do*.”

Sudden economic instability such as losing a job or separating from a partner added another layer of economic stress. Steph, a 32-year-old Kansas woman who previously had an abortion told us: “*I’ve been unemployed for a very long time, about six months. So, I felt kind of a lot of pressure in terms of money. It would cost me $1000, and I just didn’t have the* funds. *I was looking online, and I found Aid Access*.” The combination of cost and possibly facing protestors at the clinic further deterred Steph: “*It was financially, emotionally, everything…I just really…didn’t want to go back there*.” When the cost of the clinic led her to seek other options, the privacy and comfort of the service solidified this choice for her, as she explained: “*I just felt like doing it at home on my own. I felt more comfortable about doing that*.”

In Louisiana, Beth had just been fired from her job, and had recently started a new relationship. She described herself as “*very broke*,” telling us: “*I don’t have any money, I just got fired, I’m not with a partner that I think I would want to have a kid with*.” Like Steph, Beth had had a previous abortion and was familiar with medication abortion: “*This past time that I found out I was pregnant, I did a bunch of Google searches…it was saying that it was the same as the pill that you get in the clinic, and all of a little bit more concrete information…poured over that for a few hours, and I talked with my partner about it, and then pretty immediately made the decision*.”

Nicole, a 25-year-old working mother of two living in Tennessee, described tumultuous circumstances as well, explaining: “*I’m recently split from my husband, who was … he took care of me. I was completely dependent on him … I’m a single mother, I’m low income and I couldn’t really afford to even go to a facility*.” Similar to Steph, the combination of cost and the intimidating presence of protestors prompted Nicole to seek other options. When she discovered the price of the service, she thought: “*This can’t be right. There’s no way that I can get these for $90…I saw all of these stories of people who had used it safely and it worked for them, so I decided to go through with it*.”

Participants experiencing the chronic stress of living paycheck to paycheck were uncertain of how to pay for medical bills associated with pregnancy confirmation and abortion care. Some called abortion fund organizations, consulted with clinic financial advisors, or turned to their state Medicaid authority. While navigating childcare logistics and coursework, Joelle tried negotiating with clinics: “*I called everybody to try to get help with the price because I’m not rich by no means and I’m in school and I’m working and I’ve got a baby. It was very difficult. I thought it was just not going to happen*” After calling several clinics, both in her state and in neighboring states, she found: “*I think the lowest doctor who’d get the price down was $500 and I was expecting two or three. I could probably swing that, I would borrow it or do something, but $500 in a matter of a week and a half or two is just hard*.” Factoring in additional costs of childcare and travel expenses, she looked online for other options and found news reports about the telemedicine organization. Although nervous about the medication, she contacted the organization, telling us: “*Yeah, I can try this and hope it works or there’s nothing else I can do*.”

Kendra, a 29-year-old Missouri woman went to her local Planned Parenthood for her pregnancy confirmation and discussed the price of an abortion with clinic counselors: “*I gave them my income, I even showed my pay stubs so that they could see my income and even with that, it would have cost me like $450 total and to me that’s rent. Either I would have to do that or I’d have to pay rent and I have a young child, so I needed to pay rent instead*.” She knew of the telemedicine service from a previous experience with a friend and decided to pursue this pathway for herself: “*I felt really relieved when I reached out to them and they said that it was available to me, but initially it was panic. It was like I have a kid that’s under two, I have a part time job, and [I’m] really not in a good situation medically*.”

This sentiment of choosing online telemedicine due to financial hardship was especially echoed by those who were uninsured. For people who wanted an ultrasound or check-up with a doctor, lacking insurance prompted them to seek alternative routes to finding baseline reproductive care. Carmen, a 31-year-old Texas woman, was between jobs, and without insurance relied on emergency room visits for her pregnancy confirmation while she tried to apply for insurance: “*I was on the phone with insurance with the state of Texas to try to get me into a gynecologist to try to see what’s going on*,” but struggled with finding accurate information from the state on what services they cover. When one of her politically active friends posted an Instagram story about the telemedicine organization, she contacted them because it “*seemed a lot easier*” than going to a clinic.

Treecie had been recently laid-off, and explained: “*I didn’t have insurance, so I didn’t really have a choice, because even planned Parenthood, they’re not free…they’re going to charge you something*.” She couldn’t afford to pay-out-of-pocket and decided to look online for help. After reading about the organization and watching videos on YouTube of how to use medication abortion, she ordered the medication. To feel comfortable self-managing, she wanted an ultrasound to date her gestation, but because she was uninsured had a free ultrasound at a Crisis Pregnancy Care Center. After receiving confirmation of her pregnancy at 5 weeks, she went home and used the medication.

Kyla, a 28-year-old woman in South Carolina, described having no money in her bank account, experienced recent family loss, and a major transition as she moved to a new state. She recalled the frustration of trying to access healthcare without insurance when she first suspected she was pregnant. Kyla wanted to have a check-in with a physician before having an abortion. She said: “*Unfortunately, because I was unemployed, I could never follow up and actually go to a doctor to get an ultrasound or anything like that, so I had to rely on just a pregnancy test*.” Next, she explained: “*I did call a couple clinics, get some prices, did some things like that, but it’s extremely expensive. I tried to apply for health insurance, and I checked off that I was pregnant because they…it’s not like they knew what I was going to do. But nobody ever even got back to me in terms of the medicine*…” A friend told her about the telemedicine organization and told us: “*I was a little bit nervous, but I felt like that was definitely my best and only option*.”

These experiences of hardship hindering a person’s access to the clinic were key influences on their decision to pursue an alternative care pathway. For most, online telemedicine abortion felt like the only affordable option.

### 3.5. The financial burden of restrictive policy

In addition to personal financial barriers, participants discussed how restrictive abortion policies made in-clinic care expensive to access, influencing their decision to self-manage using online telemedicine. Laws that closed local abortion clinics forced people to travel long distances for care and state-mandated waiting periods added travel costs, and lost wages due to time off work. People were aware of the variation in state abortion laws, and some considered the cost of access in neighboring states, whereas others feared the harsh effects of upcoming abortion restrictions.

For Carmen, the 2013 Texas Omnibus Bill had closed her nearest abortion clinic, and as she explained: “*Your first instinct is go to Planned Parenthood. If I would have chosen that route, I would have had to travel 3 h or 4 h…then they do a whole-day appointment where you have to listen to the heartbeat, look at it on an ultrasound, talk to a lot of different doctors and nurses just so you could be sure of your decision…the cost would have been over $1000. No insurance would have covered it…that’s very expensive, traveling 4 h. And then we thought about the free abortion pills advertisement we saw or whatever, so we looked it up and [Aid Access] popped up*.”

After the Department of Health and Senior Services in Missouri refused to renew the license to her local Planned Parenthood, Kendra said: “*They don’t provide here anymore to my knowledge. My initial appointment with Planned Parenthood was in Missouri, but they decided that if I wanted to do the abortion I would have to go to Illinois. I would’ve had to drive at least an hour to Illinois to have it done, which again, that’s time away from my son, that’s time away from work that I really couldn’t afford*.”

In South Dakota, 34-year-old Rhiannon explained: “*The abortion laws are pretty strict and it’s hard to find, there’s only one abortion clinic and that’s about 5 h away from where I live. And it’s also pretty extensive in terms of they make you wait a three-day waiting period. So they have to give the information, then you have to wait three days and then they’ll finally prescribe you the pills and then you have to come back in two weeks after that. So it would require a lot of time off work*.” Conscious of waiting period policies and the increased cost of abortion care at later gestations, she told us once she found the service, she immediately started her online consultation.

The interconnected concerns of financial hardship and stressful policy contexts meant people were operating on the margins of significant risk and uncertainty, prompting them to have a back-up plan in case the pills did not arrive. Living in Louisiana, Laura felt she needed a plan in case the medication from Aid Access did not come: “*In Louisiana things are very restrictive. You have to make a minimum of two or three appointments before you can even get to the procedure. I was dealing with appointments that would have been scheduled weeks apart, at which point things would have been far enough along in the pregnancy…and so I was researching, trying to figure out how do I drive to Florida?*” She worried about how to financially and discreetly access care on the off chance the medication did not arrive: “*I just felt like I had no possible options being in the situation I was in and also, being in Louisiana…I made up this whole convoluted story to my partner about I got a ticket in Florida and I have to go back and address that and it’s too far to drive so could you lend me money for the hotel? I was going to sleep in my car…I started panicking so badly that the pills would not get here*.”

Other participants were concerned about proposed abortion restrictions they had read about in the news. Participants in Southern states mentioned seeing articles shared on Facebook about the June 2019 heartbeat bill introduced in Georgia, expressing concern that the similar bills would pass in their states. In Ohio, 21-year-old Toni was worried about providing for her 2-year-old son and wondered if she was ready for another child. After reading a research study about online telemedicine she began researching her options, but the tipping point for her was the proposed Ohio heartbeat bill: “*We have an abortion law going into effect, where if you have a heartbeat with the baby, then you can’t even get an abortion, and Planned Parenthood is booked until the day that even happens. And they’re really expensive. It’s 200 for the first appointment, and then 300 or 400 for the second appointment, and I don’t have that type of money. So I was like, ‘I need to find another way*.’ ” She contacted Aid Access: “*I was asking them about the price and stuff. I paid them…so I was just waiting and I was still searching other ways…I just kept thinking in the back of my head, ‘I hope these work*.’ ” Like Laura, Toni was nervous about receiving the medication. While she waited, she researched self-managing with raw papaya or pineapple, or ordering pills on the “*dark web*.” When the pills arrived, and she told us she felt comfortable taking them because the telemedicine organization provided detailed instructions and they “*just seemed like they cared*.”

When finances were tight the prospect of medication not arriving was a serious concern. Participants had a heightened awareness of economic and legislative constraints, and the prospect of not being able to afford to travel, or the possibility of losing $90 on a failed request for medication, was distressing for participants.

### 3.6. Navigating the economic context of motherhood

Adding to these interrelated economic and policy circumstances, a key theme among participants with children was their identity as mothers, and the responsibility they felt for their family. Toni further described to us a complicated balance of motherhood and school as she considered her options: “*I already have a child. He is about to be two, and I don’t have … I barely make enough to even survive right now. And I’m in the process of trying to move, figure out where I’m about to go, and I’m also in school. So, it’s already a lot dealing with my son, and I was concerned like I’m not going to be able to provide for both of them or handle it while I’m at school. I was afraid I would probably drop out, because last time when I was pregnant with my son, I almost dropped out of school, and school is really important to me…it’s just not the right time for me to have a child. That’s what I was thinking, so I needed help and I was like: ‘I need to figure it out now*.’ ”.

Similar to Toni, participants who already had children described an additional financial calculus as they considered the economic wellbeing of their family. Their role as a mother was intrinsically linked to the personal financial context determining which abortion care pathway they pursued. For women who had a recent birth, the financial and physical strain added an additional dimension to their decision to seek abortion care. As Joelle explained: “*I had just recently given birth in January to my now seven-month old baby and I’d been off work for probably about three months afterwards. Any savings that I had had been depleted. I’m still learning how to be a mom and then I found out I was pregnant again…It wasn’t something that I was ready for…I had just started back to work…I didn’t really have any extra money. Babies take a lot of money*.” After ordering the medication she told us: “*Ultimately [I] thought if it doesn’t work, I guess I’ll just have the baby. I don’t know. To me it was the better decision [Aid Access] for myself and for my family. I ultimately just bought the pills and hoped for the best*.”

Morgan, a 27-year-old mother of three, had recently had an emergency c-section, and due to recently diagnosed polycystic ovarian syndrome had a 7-mm cyst removed from her right ovary. She described her pregnancy as: “*That whole – everything was just so traumatic, and getting pregnant so fast and so suddenly, it was terrifying. I had just started a new job. I had just gotten a promotion. I already have three kids. It was not the right time*.” After being turned away from care by a pro-life gynecologist, she went to Planned Parenthood to confirm her pregnancy, cautious because of an ectopic pregnancy she had in the past: “*They checked me…and then they told me how much it was going to cost – it already cost me $180 just for that appointment, and then they told me it was going to be another $580 for just the pills*.” Morgan took some time to consider the price of in-clinic care, concluding: “*There’s no possible way. I literally just had a baby. I’m buying diapers. I have two other kids at home. I don’t get any child* support *from their dad. I have to help my parents pay bills. I’m struggling. There’s no way I can just pull 500 bucks, 600 bucks out my butt*.” In passing an acquaintance told her about the telemedicine organization, and after doing some research she ordered the medication. After using the medication at-home she stressed: “*The price point was a lot cheaper than here in the United States, and that’s why a lot of people are forced to continue their pregnancy, because it is so expensive*.”

For Toni, Joelle, and Morgan, it was clear to them that the economic demands of their family made it impossible to have another child, let alone the unexpected expense of an in-clinic abortion. But for other mothers, the economic responsibility of their family elicited more complicated feelings. Some wanted to grow their family but felt conflicted about providing for their current children as the first priority. Liz, a 31-year-old mother of three in Wisconsin, found out she was pregnant after taking an at-home test and when we asked about her decision to end her pregnancy, she put it simply: “*It would take away what I can buy for my current kids. They would suffer*.” She thought about traveling to Mexico for pills, but then she found Aid Access: “*The only place I found to get them is either the [Aid Access] website or Planned Parenthood. I couldn’t find anywhere else. Those are it, you know*.”

As Laura spoke with us, she discussed the joy her daughter brought her: “*I feel like I’m looking at this beautiful little five-month old baby and I’m like she’s wonderful. I would love to have another one just like her,’ but…I think about her first and if I have another one financially, I don’t know that I can support two at this point and be pregnant and go to work and everything else*.” Laura was struggling with a recent job loss and the passing of her sister: “*There’s a large part of me that wanted very much to have a child. Then there was obviously another part of me that said what in the hell am I going to do trying to have this child in the middle of everything I’m in right now? I certainly, again because of my employment situation and emotional state, wasn’t in a position to do that by myself*.”

Andrea, a 30-year-old mother in Arizona, considered having another child, and expressed complicated feelings about growing her family: “*Just because like I didn’t … I figured like because I have two kids…it would be selfish of me to make that decision knowing that we couldn’t handle another child. And forcing another thousands of dollars in expenses that we couldn’t afford…it is just already hard to make ends meet*.” Consistent among the mothers we spoke with, in the end their family’s financial wellbeing was a major factor in their decision to self-manage. They expressed love and gratitude for their current children, as well as a commitment to doing the best they could for their family’s economic wellbeing. Many mothers who have in-clinic abortions likely also struggle with these difficult decisions, but among the mothers we spoke with, the key factor leading them to use online telemedicine was cost. Further guiding them to this option were the themes echoed by all our interview participants, that self-managing with online telemedicine offered flexibility and privacy, and with the added time and logistical constraints of motherhood, the convenience of telemedicine was especially important. This pathway was a way to ensure they could fulfill their commitment to their family and access the care they desired.

### 3.7. Even a little is too much

When participants experiencing financial hardship discovered they could access medication abortion using the online telemedicine service, many expressed relief at the reduced price. Joan told us she used some of her savings to cover costs, reacting to the $90 donation as: “*I mean, it was a lot better than the $800 for Planned Parenthood. So did we feel it? Yeah. But I mean, did we make it happen because it needed to happen? Yeah*.” Like Joan, many were conscious of the additional expense, and others expressed distress when faced with the full donation amount of $90. Of the eighty participants we spoke with, 51% could not afford the full amount (Table 3). Aid Access operates on a sliding scale suggested donation system, and because of their financial situation some women qualified to receive medication at a free or reduced cost. Deidre, a 30-year-old mother of three in South Carolina told us: “*At the time, I didn’t even have the funds that they were asking for. I think it was 90 dollars. Of course, I knew the longer you waited, the higher the chances are that I would pass the [gestational] window, and I was just honest about my current situation and they were able to get me free access to the meds I needed*.”

Alexandra, a 20-year-old mother of two living in Texas described: “*I emailed them and I told them, ‘Is there any way I can lower the price down?’ And they were like, they emailed me back the same day, and said, ‘Yes. What would work for you?’ So then I told them, ‘I only have $40.’ And that’s what I sent them*.” At the time her boyfriend was working while she cared for her two children. She struggled with how to talk with him about her decision to have an abortion, let alone ask him to help her cover the cost of donating. She explained: “*So if I was to tell him, ‘Can you give me $80?’ He’d be like, ‘Why would you need $80? We have two kids, we have stuff to buy, we have diapers to buy…he even said, ‘Well, we don’t have $700 … well, we do but we have a truck payment so how are we going to do that? We’re going to miss the truck payment or are we going to get an abortion?*’ ” Ultimately Alexandra’s boyfriend helped her with the donation, and she ordered the medication. Like Alexandra, several respondents asked people in their networks to help them with the donation, but privacy concerns and abortion stigma prompted other women to work extra hours or earn money from a side job. Steph worked after hours delivering food for Door Dash, while Morgan sold makeup palettes at a garage sale, and 23-year-old Rosie sold her artwork in her rural Oklahoma town. Although the decision to use online telemedicine was often motivated by being unable to afford in-clinic care, even the telemedicine service itself was a struggle for some to afford.

## 4. Discussion

These findings demonstrate that self-managed abortion using online telemedicine offered an affordable alternative to the high costs of in-clinic abortion care. Interview participants expressed intersecting experiences of personal financial hardship and restrictive abortion policies, prompting them to seek an alternative pathway to care. Additional financial considerations such as pregnancy confirmation, mandatory ultrasound and waiting periods, traveling for appointments, securing childcare, or finding transportation, compounded the financial burdens populations with low incomes already face. An added layer of complexity in these interviews was the role of motherhood in decision-making. Many mothers had complicated feelings of perhaps wanting another child yet holding a candid understanding of the economic strain of a growing family. Mothers especially discussed the difficulties of navigating an intricate balance of work, education, and familial obligations while seeking abortion care. For several interconnected reasons, Aid Access offered an economically feasible option. Yet for some, affording even the $90 donation requested by the service was a financial struggle.

This study makes an important and novel contribution to the literature on self-managed abortion and abortion access for populations with low incomes. While some scholarship has examined knowledge of and interest in self-managed medication abortion in the U.S (Aiken et al., 2020a; Grossman et al., 2015), our study is the first to examine how financial hardship leads people to self-manage using online telemedicine and shapes their self-management experience. Furthermore, by focusing on the economic necessity of access to telemedicine abortion, we examine a population in the U.S. frequently left without access. As restrictive laws continue to impact the availability of abortion in the U.S., these findings are especially important to consider for people’s ability to seek and obtain care that meets their personal preferences and allows them to maintain their economic security (Miller et al., 2020).

Researchers and advocates are optimistic about self-managed medication abortion as an innovative pathway for equalizing abortion access (Donovan, 2018; Jelinska & Yanow, 2018). In legally restricted settings, self-managed abortion is regarded as expanding access to abortion care (Gomperts et al., 2008; Kapp et al., 2018), and meeting preferences of convenience, privacy, and cost (Aiken et al., 2018a). Clinic-facilitated telemedicine and for-profit telemedicine models have recently formed in the U.S. with the goal of expanding access to abortion and serving populations who cannot reach the clinic. Our analysis of data from a non-profit, sliding scale service, challenge the notion that self-managed abortion using medication abortion will accomplish full accessibility in populations with low incomes. The critical piece here is not simply access to medication abortion, but access to services that people can afford. Our interview participants were candid in describing the complexities of their lives, the intersecting effects of personal financial hardship and policy barriers, and the careful considerations they made for themselves and their families to stay afloat. The inability to afford in-clinic pregnancy confirmation appointments, and the reality of insurance restricted access to care are evidence of systemic failures in our healthcare system. The constraints of motherhood on logistics highlight how complex access to reproductive care can be when caring for a family. We find evidence that self-managed abortion provided an affordable alternative to the clinic, and at times even a preferential pathway for seeking abortion care. However, the inability of our study population to sustain the burden of associated pregnancy costs and at times even the full cost of medication exemplifies that the online telemedicine organization still posed a financial burden. These findings highlight the broader reality that for those already struggling financially, unexpected healthcare expenses, even if significantly reduced from the typical price, are still too much.

It is also important to note that while self-managed abortion was a safe and acceptable option for the individuals in this study, for others in-clinic abortion care may meet personal preferences and needs. There must be a wide range of abortion care options in the U.S. that are accessible and acceptable, and protecting clinical abortion access is essential for achieving this. Many of the economic burdens to clinical care discussed in this study and in the literature can be alleviated by ending the Hyde Amendment, and expanding clinical abortion coverage to the estimated 13.9 million women of reproductive age who are insured through the Medicaid program (Kaiser Family Foundation, 2021).

### 4.1. Limitations and strengths

The primary limitation of our study is that our sample was necessarily self-selected, and therefore our results may have limited generalizability. Our analysis was limited to those seeking access to medication abortion up to 10 weeks’ gestation, omitting the experiences of those seeking care at later gestations. There are other methods of self-managed abortion not using medication abortion or telemedicine, and these are not captured in this study. A strength of our methodology is it illuminates the range of experiences of abortion patients in the U.S. who receive care outside of the formal healthcare setting and source alternative pathways to accessing abortion. This population is difficult to access and not captured with typical clinic-based methodologies, and this unique data offers an opportunity to understand the experiences of this hard to reach population.

### 4.2. Conclusion

A person’s economic security acts as a major determinant of how they access to abortion care. As abortion restrictions continue to pass at the state level and federal intervention remains uncertain, it is likely that abortion access will only become more difficult for populations with low incomes. Public policy interventions are an opportunity to address these economic disparities in access to care. Repealing the Hyde Amendment and expanding both public and private insurance coverage for abortion care will increase financial access to the clinic (Ibis Reproductive Health, 2017). Re-evaluating the Food and Drug Administration’s Risk Evaluation and Mitigation Strategies classification of mifepristone would increase prescription of medication abortion, and the ability to facilitate medication abortion provision by clinics using telehealth models (Sixteen Years of Overregu, 2017). Evidence suggests that self-managed abortion will continue, and policy-makers can repeal laws that intentionally criminalize this practice (If/When/How, 2019). The COVID-19 global pandemic has fast-tracked innovative medication abortion telemedicine provision, and it is important to consider these findings when implementing models that are both digitally accessible and affordable. This study offers a candid look into the complex lives of individuals seeking abortion care, serving as evidence of the intricate link between abortion access and economic security. There are major opportunities for telemedicine to increase access to medication abortion, but it is important to understand this promising practice is not beyond the scope of the structural inequalities that make healthcare in the United States difficult to afford. As the field of sexual and reproductive health continues to examine self-managed abortion and telemedicine access, it is critical to consider the experiences of populations that are particularly economically fragile and frequently underserved by the U.S. healthcare system.

## Figures and Tables

**Table 1:** Self-reported demographic characteristics of interview participants who self-managed their abortion using medication from an online telemedicine service (n = 80).

Characteristics	Frequency

**Age (years)** 18–19 20–24 25–29 30–34 35–39 40–44 Missing	8 (10.0%) 22 (26.3%) 27 (33.8%) 6 (20.0%) 6 (7.5%) 1 (1.3%) 1 (1.3%)
**Race/Ethnicity** Asian Black/African American (Not Hispanic) Hispanic/Latino Middle Eastern Mixed Race (Hispanic) Mixed Race Native Hawaiian Native American White (Not Hispanic) White (Hispanic/Latino) Other Chose not to disclose	1 (1.3%) 14 (17.5%) 12 (15.0%) 1 (1.3%) 2 (2.5%) 5 (6.3%) 2 (2.5%) 1 (1.3%) 37 (46.3%) 3 (3.8%) 1 (1.3%) 1 (1.3%)
**Gender** Female Nonbinary Missing	79 (98.8%) 1 (1.3%) 0 (0.0%)
**Sexual Identity** Heterosexual Homosexual Bisexual “Bi-curious” Pansexual “No Preference” Missing	65 (81.3%) 1 (0.0%) 9 (11.3%) 2 (1.3%) 3 (2.5%) 1 (1.3%) 2 (2.5%)
**Children** 0 >1 Missing	31 (38.8%) 49 (61.3%) 0 (0.0%)
**Employment Status** Working Working & in school Retired & in school In School Not working Missing	42 (52.5%) 17 (21.3%) 1 (1.3%) 5 (6.3%) 15 (18.8%) 0 (0.0%)

**Table 2:** Geographic location and state policy context of the state of residence for interview participants who self-managed their abortion using medication from an online telemedicine service (n = 80).

Geographic Distribution	Frequency

City	35 (43.8%)
Suburb	14 (17.5%)
Town	15 (18.8%)
Rural	14 (17.5%)
Missing	2 (2.5%)
**State Policy Context**	**Frequency**
Supportive	10 (12.5%)
Middle	0 (0%)
Hostile	44 (55%)
Very Hostile	24 (30%)
Missing	2 (2.5%)

**Table 3:** Amount interview participants donated to the online telemedicine organization for receipt of medication (n = 80).

Amount donated (U.S. dollars)	Frequency

Full donation	39 (49%)
Partial donation	29 (36%)
No-cost	12 (15%)
Missing	0 (0.0%)
